# Salinomycin, as an autophagy modulator-- a new avenue to anticancer: a review

**DOI:** 10.1186/s13046-018-0680-z

**Published:** 2018-02-13

**Authors:** Jiang Jiang, Hailong Li, Eskandar Qaed, Jing Zhang, Yushu Song, Rong Wu, Xinmiao Bu, Qinyan Wang, Zeyao Tang

**Affiliations:** Department of Pharmacology, Dalian Medical University, 9 west section, south road of Lvshun, Dalian, 116044 China

**Keywords:** Salinomycin, Autophagy regulator, Preference for cancer stem cells (CSCs) or cancer, Anticancer agent

## Abstract

Since Salinomycin (Sal) emerged its ability to target breast cancer stem cells in 2009, numerous experiments have been carried out to test Sal’s anticancer effects. What deserve to be mentioned is that Sal can efficiently induce proliferation inhibition, cell death and metastasis suppression against human cancers from different origins both in vivo and in vitro without causing serious side effects as the conventional chemotherapeutical drugs on the body. There may be novel cell death pathways involving the anticancer effects of Sal except the conventional pathways, such as autophagic pathway. This review is focused on how autophagy involves the effects of Sal, trying to describe clearly and systematically why autophagy plays a vital role in predominant anticancer effects of Sal, including its distinctive characteristic. Based on recent advances, we present evidence that a dual role of Sal involving in autophagy may account for its unique anticancer effects - the preference for cancer cells. Further researches are required to confirm the authenticity of this suppose in order to develop an ideal anticancer drug.

## Background

According to the latest World Health Organization (WHO) data, cancer is the second-leading cause of death globally and accounts for 8.8 million death in 2015 [1]. However, major current tumor therapeutic strategies like operation, radio- and chemo-therapy still exist some defects, failing to cure most tumor patients completely. Cancer stem cells (CSCs), which are resistant to many current anticancer therapies, perhaps account for the failure of treatments. CSCs refer to the subpopulation of cancer cells endowed with self-renewal, multi-lineage differential capacity and innate resistance to conventional radio- and chemo- therapy [2]. CSCs, relying on those capacities, are regarded as the culprit of recurrence and metastasis of cancer [3, 4]. Hence, eradication of CSCs will be the key to the success of cancer treatment.

**Fig. 1:**
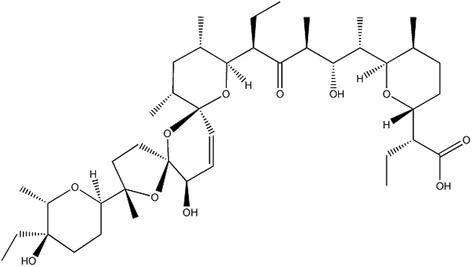
The structural formula of Sal. Sal is a 750 Da monocarboxylic polyether antibiotic with unique tricyclic ring system, whose molecular formula is C_42_H_70_O_11_

Of note, with further study of Sal, it stands out as one of the notable landmarks in the progress of chemotherapeutical drugs on CSCs. Sal, isolated from the bacterium Streptomyces albusin 1974 [5](See Fig. 1), exhibits a broad-spectrum antibiotic activity particularly against Gram-positive bacteria, fungi, parasites, protozoa [5, 6]. It is widely used as an anticoccidial drug in animal farming and is fed to ruminants to improve nutrient absorption and promote growth [7]. In 2009, Gupta et al. screened about 16,000 compounds in order to hunt for chemicals that are preferentially toxic to CSCs. The screening identified 32 substances that are able to impair CSCs. Finally, Sal was found to be the most efficient one, having a more than 100-fold efficiency of Sal compared to Paclitaxel to kill breast CSCs in mice [8]. After that, the efficiency of Sal against the CSCs in several malignancies, including breast-, prostrate-, brain-, blood-, liver-, pancreatic-, skeleton- and lung cancers have been further verified [9–12]. In addition, it has been proved that Sal is able to kill chemotherapeutical agents resistant cancer cells such as Doxorubicin-, Cisplatin-, Gemcitabine-, Temozolamide-, verapamil- and Imatinib- resistant cells and simultaneously sensitize radio-resistant cancer cells [9, 13–15]. Besides its predominant anticancer activities, it has been also verified that Sal does not emerge severe adverse effects on human normal tissues like other conventional chemotherapeutical drugs. Sal induces T-cells apoptosis in T-lymphocytic leukemia patients, but not in healthy people [16]. Similar results have been demonstrated in further studies [17, 18]. Furthermore, several successful pilot studies in cancer patients have showed temporary and minor effects while causing the regression of various solid tumors [9, 19].

Despite the predominant antitumor effects and fewer adverse effects of Sal, the mechanism by which Sal brings about cancer cell death while non-malignant cells are exempted from the lethal effects remaining poorly understood. According to the studies both in vivo and in vitro, such following mechanisms that mitochondria-dependent cell death [20, 21], Death receptor - mediated cell death [14], increased DNA damage and cell cycle arrest [22, 23], p-glycoprotein inhibition [24, 25] have been reported to involve the predominant anticancer effects of Sal. However, those mechanisms may just play a partial role in the anti-cancer effects of Sal as conventional chemotherapeutical agents rely on them to induce cancer cell death. Further studies have demonstrated that Sal suppresses Wnt/β-catenin signaling pathway conferring CSCs resistance to radiation [26, 27] and to chemotherapeutical agents [28, 29]. Moreover, other studies have showed that Sal blocks the Hedgehog (Hh) pathway, which plays a crucial role in the stemness maintenance of CSCs [30, 31]. Those may elucidate Sal’s preferential toxicity towards CSCs. Besides the classical cell death pathway, alternative cell death pathway has been also observed in the experiments, the result has displayed that Sal is able to mediate apoptosis in cancer cells through a pathway independent of activation of p53, CD95/CD95L system, caspase, and the proteasome [32]. A variety of cell death pathways or the apoptotic signal pathways forming a complex reticular structure involves in the anticancer effects of Sal, but the key component to initiate that process is still unknown, what a role autophagy plays in the reticular structure requires more studies.

Conventional chemotherapeutical drugs cannot efficiently kill CSCs, which led to the recurrence of the cancer [4, 33, 34]. Comparing to the conventional chemotherapeutical drugs, Sal targeting CSCs must have its unique targets or signaling pathways. Therefore, the study of Sal cannot only provide an effective tumor treatment drug, but also can deepen the understanding of the characteristics of CSCs, so as to give a basis for the development of new anticancer drugs, and ultimately overcome this catastrophic disease.

With a further research, scientists have found that autophagy is a homeostatic process that prevents the accumulation of impaired proteins and organelles and limits the production of reactive oxygen species (ROS). Autophagy can simultaneously provide the cell with nutrients and energy that it needs to survive. However, autophagy plays multifarious roles in the initiation, development and progression of cancer. On the one hand, autophagy suppresses malignant transformation by many mechanisms such as maintaining genomic stability via the elimination of dysfunctional mitochondria and the decrease of ROS production, the degradation of onco-proteins, the induction cell death following oncogene activation, anti-inflammatory function and assisting the immune system to eliminate the malignant cells [35]. On the other hand, after a formation of neoplastic lesion, autophagy is believed to promote and maintain tumorigenesis and to mediate resistance to different forms of anticancer therapy [36]. Autophagy is generally considered to play a cyto-protective role for cell survival against apoptosis. However with further study, many literatures have demonstrated that autophagy is primarily a cell survival pathway, but if excessive, it may kill cells [37–39]. So the exact role of autophagy playing in the death process of cancer cells requires further study. Additionally, autophagy is dysregulated in various diseases, such as neurodegenerative diseases, immune system diseases and cancers.

## Sal and Autophagy

### Sal is an inducer of autophagy

Similar to other chemotherapeutical agents, the administration of Sal in the tumor cells induces autophagy. It was firstly discovered that Sal induces autophagy in colon and breast cancer cells [40]. The vast majority of autophagy has a protective effect in all tested cancer cell lines. However, Sal-induced autophagy in SW620 cells is involved in provoking cell death, as inhibiting autophagy through transfection with siRNA against ATG7 as a result of partially preventing autophagy induced cell death in 24 h by inhibiting autophagy. Pre-treatment with the PI3K inhibitor, wortmannin, which is commonly used to inhibit autophagy, gets an isovalent effect [40].

Relevant studies have undertaken to verify the mechanisms and roles of autophagy induced by Sal in other human cancer cell lines. Most of them have found that Sal induces cyto-protective autophagy, facilitating Sal-induced cell death after inhibiting the autophagy [18, 20, 41–43]. Combined use of Sal and chloroquine (CQ), which is an autophagy inhibitor, not only improves antitumor effects of Sal but also reduces the dose of Sal, which means that combined use of autophagy inhibitors reduces the side effects of Sal, though for now Sal’s adverse effects are slight and transient [44]. Although autophagy plays a cyto-protective role in most cancer cell lines, autophagy also can be an alternative cell death way in a small part of the cancer cell line as described above. So whether the combined use of Sal and autophagy inhibitors being beneficial or not are deserved to be further studied in various human cancer cell lines before Sal is used formally in clinical anti-tumor therapy.

### Sal is an inhibitor of autophagy as well

**Fig. 2:**
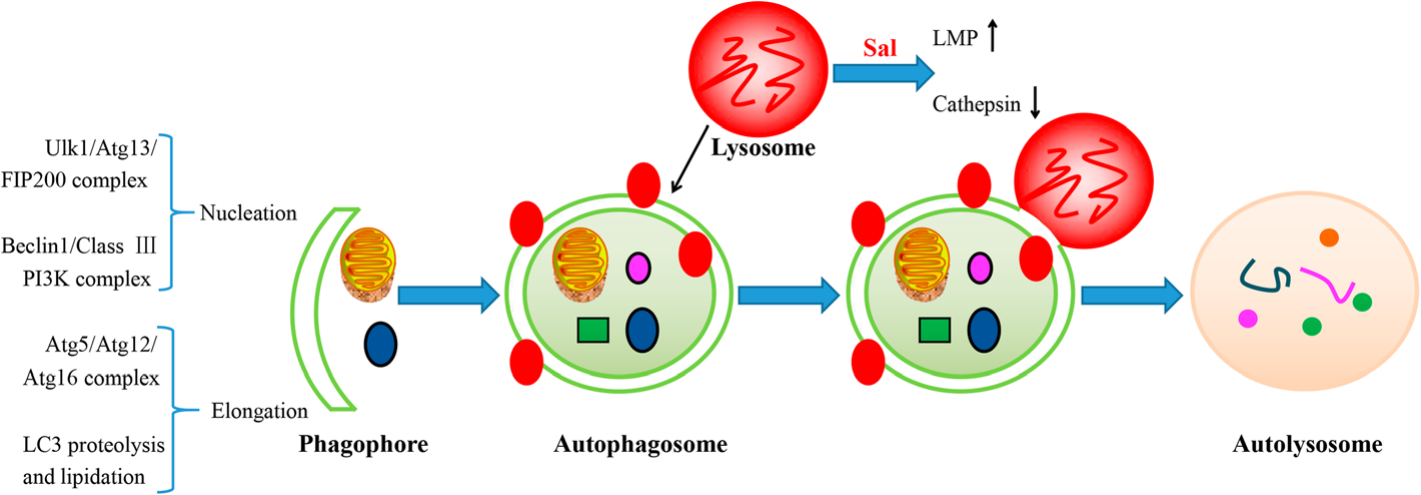
Diagram illustrates the positions of Sal’s inhibition of autophagic flux. Macroautophagy begins with engulfment of the cytoplasmic materials by the phagophore, which detains the materials into a double-membrane vesicle (i.e. autophagosome). The autophagosome fuses with a lysosome to form an autolysosome, and then the contents are degraded by the lysosome. Initiation of autophagosome formation requires the ULK1-Atg13-FIP200 complexes and Beclin1-class III PI3K complexes. Two conjugation systems, Atg12-Atg5-Atg16 and LC3, are critical to the elongation and enclosure step of the autophagosome formation. Lipid conjugation leads to the conversion of the soluble form of LC3-I to the autophagic vesicle-associated form LC3-II, which is widely used as a marker of autophagy. Sal is found to increase the lysosome membrane permeability (LMP), which affects the acidification and the integrity of lysosomes. Moreover, Sal can also inactivate cathepsins in lysosomes, resulting in the inhibition of lysosomal activity

Autophagy is a homeostatic process that prevents the accumulation of impaired proteins and organelles and limits the production of ROS, lessening the impairment to cells. It was discovered interestingly that Sal can inhibit the autophagic flux in breast CSCs interfering with their stemness maintenance. Moreover, Sal does not impair the fusion of autophagosomes and lysosomes. Besides, Sal has no effect on the pH of lysosomes. Using an in vitro cathepsin substrate assay, it has been further demonstrated that Sal dramatically reduces the activity of cathepsins, resulting in the inhibition of lysosomal activity. Sal’s inhibition on autophagy may account for that Sal can block the degradation of mCherry-GFP-LC3 and long-lived proteins [45]. The study has finally revealed that Sal inhibits the autophagic flux in cancer cells by inhibiting the lysosomal activity via inactivation of cathepsins without altering the integrity of the lysosomal compartment [45]. Furthermore, another study discovered that Sal induces an aberrant autophagic flux via the production of ROS in glioblastoma as the alleviation of ROS production restored the autophagic flux. In addition, the inactivation of cathepsin B, Sal also increases the lysosome membrane permeability (LMP) through lipid oxidation, which is confirmed by the release of cathepsin B from lysosomes (See Fig. 2). Both lead to the blocking of autophagic flux. However, a study has found that Sal can block acidification as a result of that the increase of ROS levels can alter lysosomal lipids, affecting lysosome-autophagosome fusion. However, another study has showed a contradictory result [46].

Moreover, a new study has displayed that Sal suppresses late stages of autophagy, leading to impaired recycling and the accumulation of dysfunctional mitochondria accompanying increased ROS- production. All of which are associated with induction of apoptosis. And that the effects of Sal are dose- and time- dependent manner and could be readily replicated by pharmacological or genetic inhibition of Hepatocellular Carcinoma (HCC)-autophagy alone [47]. It has been reported in different animal models that the tumor extracellular pH (pHe) ranges are between 5.9 and 7.2 [48, 49], with average tumor pHe often reported ~ 6.5 [50, 51]. A study has discovered that unlike CQ and its derivatives whose activity are to different degrees when counteracted by acidosis, the ability of Sal to inhibit autophagic flux is potently enhanced in different conditions of both transient and chronic acidosis [52]. Such activity may be linked to the physicochemical characteristics of Sal, which is a weakly acidic, while lipophilic compound with a reported pKa of 6.4 due to its carboxylic group [53]. Moreover, a study also has demonstrated that tumor-mimicking micro-environments can enhance the action of Sal. Furthermore, hypoxic, low-glucose and low-nutrient conditions in hypo-perfused tumor, actually are able to potentiate Sal’s toxicity, and to increase its preferential anticancer activity [52].

Generally speaking, the ability of Sal to induce or inhibit autophagic flux has been verified in various human cancer cell lines. For example, Sal can induce functional autophagy at lower concentrations and such a dose is cell type-dependent. Interestingly, PC3 cells show active autophagic flux at 10 μM concentration of Sal while murine embryonic fibroblasts already show an inhibition of autophagic flux at such doses. A higher concentration of Sal (i.e. 30 μM) inhibits autophagic flux in both cell types. This result has manifested that Sal is an inducer of autophagy, whereas autophagic flux inhibition is a secondary response [54]. Similarly, 10 μM Sal treatment of MCF7 cells for 16 h downregulated Beclin1 and ATG12, which are involved in the initiation and elongation of autophagy, while showing an increase at initial time points (2~ 8 h) and lower doses [40]. Whether the autophagic inhibition of Sal exists in all cancer cell lines demanding further confirmation. And whether there are additional mechanisms besides the increase of LMP and inactivation of cathepsins equally worthy of study. Therefore, when using Sal, it should be determined its suitable doses when Sal acts as an autophagic inhibitor in various tumors and induces cancer cells death simultaneously so as to provide a rational guide for dose selection of clinical treatment of tumors.

## The conceivable interpretation for Sal’s preference for cancer cells or CSCs

Chemotherapeutics currently used kill tumor cells but also cause impairment to human normal tissues, such as myelosuppression, immunosuppression, nephrotoxicity, liver injury. Interestingly, Sal selectively induces cancer cells death while sparing benign cells. This may be related to Sal’s distinct targets or signaling pathways that are lethal to cancer cells while are slighter to human normal tissues. Here we will explore whether the inhibition of autophagy is one of the explanations for Sal’s preference for cancer.

**Fig. 3:**
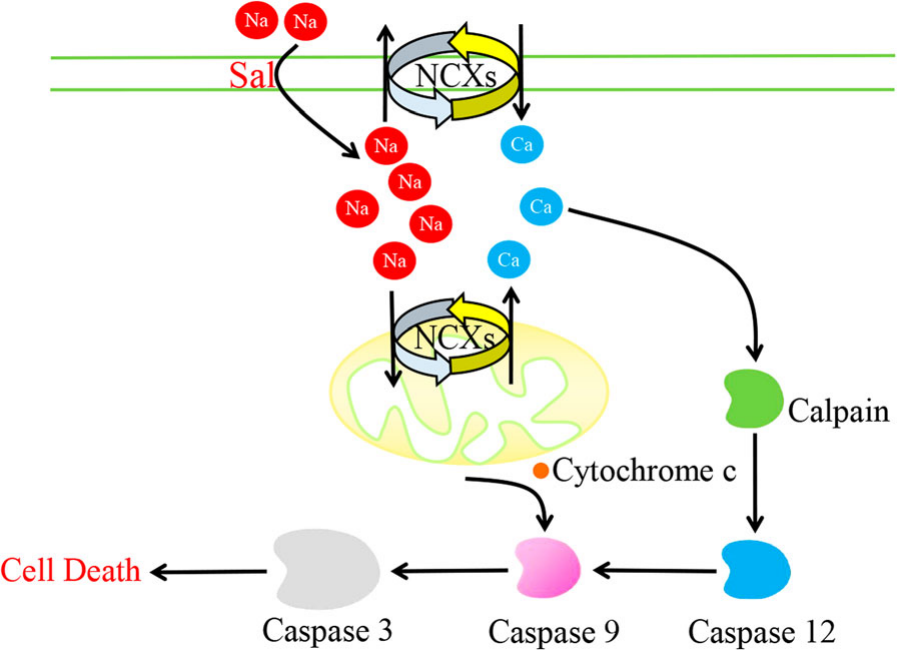
Diagram illustrates the observed effects of Sal-induced toxicity in DRGNs. Sal triggers an increase of cytosolic Na^+^ concentrations, with a secondary increase in cytosolic Ca^2+^, which is mediated by NCXs in the plasma membrane and mitochondria. Elevatory intracellular Ca^2+^ levels activate the protease calpain, which leads to apoptotic cell death by the activation of caspase pathways involving caspase 12, caspase 9 and caspase 3. Caspase 9 can be alternatively activated by cytochrome c released from depolarized mitochondria

Mitochondria, generally called the powerhouses of the cell, comprises a dynamic interconnected network of tubular structures that are engaged in fission and fusion processes [55]. Mitochondrial fission is mediated by localization of dynamin-related protein (Drp-1) on the mitochondrial site of division whereas fusion is mediated by mitofusin proteins (mitofusins 1 and 2) along with optic atrophy protein 1 (OPA1) [56]. Lack of both mitofusin 1 (Mfn1) and mitofusin 2 (Mfn2) results in damaged mitochondrial fusion, but both of them can compensate each other’s deficiency [57–60]. Facing stress or any event leading to dysfunction of mitochondria, the organelle undergoes an asymmetric and protective fission that aims to spare at least part of stressed mitochondria, by cleaving the organelle into a normally-functional part, and a dysfunctional one. A recent study has described that cancer cells often have much smaller, fragmented mitochondria in comparison to normal cells. In the same study, the results have showed that cancer cells express an increased level of Drp1 and decreased level of Mfn protein, leading to a constant mitochondrial fission with impaired fusion, resulting in a smaller fragmented mitochondria in cancer cells [61]. Another study has displayed that specific toxicity of Sal to cancer cells is through mitochondrial hyperpolarization that is observed preferentially in cancer cells. Moreover, Sal-triggered depletion of cellular ATP, in cancer cells but not in primary cells, contributes to Sal’s preferential anticancer toxicity to cancer [62]. Furthermore, Sal induces depolarization of mitochondria contrasting to Dichloroacetate (DCA), which is also a K^+^ ion channel modulator, and a molecule that also preferentially targets cancer cells [63, 64]. The observed cancer cells specific deleterious effect of Sal is explained by the fact that cancer cells harbor various abnormalities within mitochondria, such as hyperpolarized mitochondria along with defective mitochondrial fission and fusion mechanisms due to the lack of functionally intact mitofusin proteins, DRP1 and other proteins involved in the mitochondrial dynamics [61, 63]. Similarly, a recent study has pointed out that Sal in concentrations effective against CSCs exerts profound toxicity towards both dorsal root ganglia as well as Schwann cells. With further study, the result has showed that the toxic effect is mediated by elevated cytosolic Na^+^ concentrations, which in turn causes an increase of cytosolic Ca^2+^ by means of Na^+^/Ca^2+^ exchangers (NCXs) in the plasma membrane as well as the mitochondria membrane. Elevated Ca^2+^ then activates calpain, which triggers caspase-dependent apoptosis involving caspases-12, − 9 and − 3. Combined inhibition of calpain and the mitochondrial NCXs result in significantly decreased cytotoxicity as compared to caspase-3 inhibition in vitro [65]. Furthermore, co-treatment with an inhibitor of the mitochondrial NCX can significantly reduce structural damage in the peripheral nervous system without impairing Sal’s antineoplastic efficacy in vivo [66]. This may account for the abnormal mitochondria in cancer cells, which is not resistant to the effect of Sal. Fig. 3 shows the conceivable mechanism by which Sal damages peripheral nervous system.

Emerging data indicate the crucial role of autophagy in the survival, self-renewal and differentiation of both stem cells and CSCs [67–69]. Recent study has found that the basal autophagic flux is higher in the ALDH(+) population derived from the HMLER cell line than in the corresponding ALDH1(−) population, the former owns more CSCs features than the latter. Autophagic flux falls in both ALDH(+) and ALDH(−) populations following Sal treatment. However, the fall of autophagic flux is greater in ALDH(+) population than in the ALDH(−) one (51% vs. 10%, respectively). Consistent with data has showed that apoptotic cell death was higher in the ALDH1(+) population derived from the HMLER cell line than in the ALDH(−) population [45]. Similar results have been acquired by another study that the subline HMLER CD24 low, carrying CSC properties has an increased sensitivity to autophagy inhibition mediated by Sal with respect to the non-CSC subline HMLER CD24(+) [52].

Autophagy is generally considered to be vital for the elimination of dysfunctional mitochondria that may actually consume ATP, and/or generate excessive amounts of harmful ROS, thus contributing to loss of cellular homeostasis [70], thus the inhibition of autophagy will enhance the apoptosis. The elimination of dysfunctional mitochondria is highly dependent on autophagy, however, Sal can induce a profound inhibition on the late stage of autophagic flux. That is, on the one hand to induce deformed mitochondria in cancer cells to be dysfunctional, on the other hand Sal inhibits the elimination of these dysfunctional mitochondria with increased ROS-production and induction of apoptosis.

## The mechanisms of autophagy induced by Sal

**Fig. 4:**
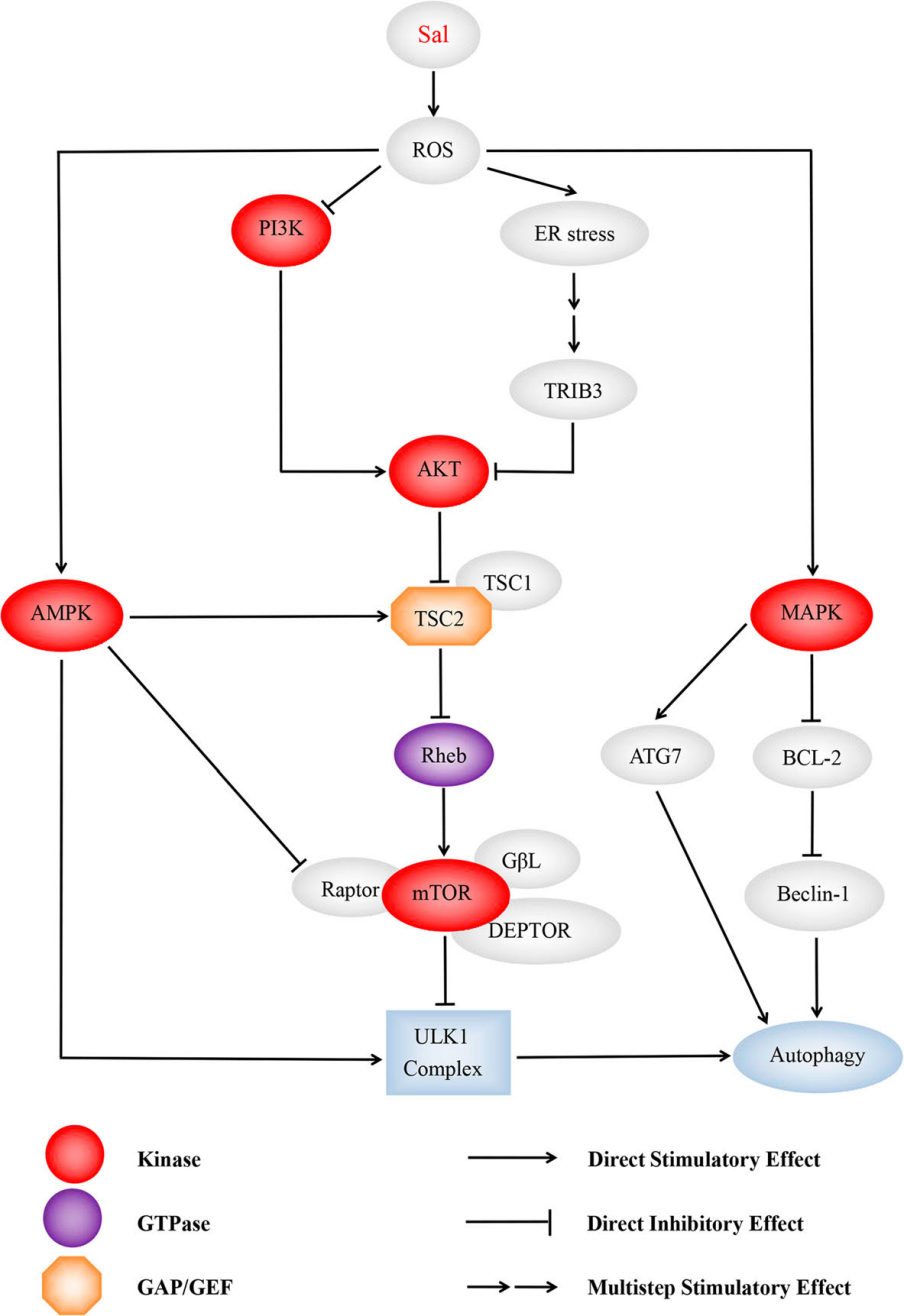
Diagram illustrates the dominating signaling pathways involving Sal- induced autophagy. Sal is able to induce intensive autophagic flux than commonly used autophagic inducers such as chloroquine, as many autophagy-initiated pathways being activated by Sal. ROS is deemed to play a key role in it. Pretreating with NAC, an ROS inhibitor, suppresses the autophagic flux being confirmed by different researchers. Sal activates AMPK signaling pathway, MAPK signaling pathway and ER stress initiating autophagy, and inhibits PI3K/AKT/mTORC1 axis simultaneously. (1) AMPK activates autophagy by directly binding and activating ULK1 complex through phosphorylation of Ser 317 [110, 111]. Enhanced stimulatory TSC2 phosphorylation at Ser-1387 by AMPK, and reduced inhibitory TSC2 phosphorylation at Ser-939/Thr-1462 catalyzed by AKT augmented TSC2/TSC1 activity, which led to mTORC1 inhibition. AMPK-mediated raptor phosphorylation further reduced mTOR’s kinase function and mTORC1 activity [42]; (2) Moreover, it has been reported that ROS suppress PI3K/AKT/mTOR signaling [112]. In addition, it’s demonstrated that Sal suppresses AKT1 activity through ATF4-DDIT3/CHOP-TRIB3-AKT1 axis in human cancer cells after activation of ER stress response, resulting in MTOR inhibition and autophagy consequently [43]; (3) ROS inactivates MAPK phosphatases [113]. This subsequently leads to the phosphorylation of JNK, and of its target the transcription factor JUN. JNK activation may promote autophagy through induction of ATG7 [94], or by phosphorylation of BCL2, which leads to dissociation of BCL2 from Beclin-1 [95]

Since the first article, reporting that Sal is able to induce autophagy in cancer cells, was published in 2012 [40], different tumor cell lines have been used to detect the mechanism of autophagy induced by Sal. Sal is a K^+^ ionophore that interferes with transmembrane K^+^ potential and facilitates the efflux of K^+^ from mitochondria [71]. Sal causes the decline of mitochondrial membrane potential (MMP) and the accumulation of ROS [20, 72]. Most of the tumor cells are found to own increased ROS after the treatment with Sal, which plays an important and complex role in autophagic activation. To clarify the role of ROS in Sal-induced autophagy, U2OS cells are pre-treated with N-acetyl-L-cysteine (NAC), a ROS inhibitor. As a result, the sharp decrease of LC3II expression and acidic vesicular organelles (AVO) accumulation is detected [20]. Similar results are also found in osteoblastoma [42], breast cancer [73], prostate cancer [74], glioma [75]. Growing data have manifested that ROS inhibits PI3K/AKT/mTOR signaling [76] and activates the AMP-activated protein kinase (AMPK) signaling [77]. ROS is also reported to initiate mitogen-activated protein kinase (MAPK) signaling, including of C-jun N-terminal kinase (JNK), p38 and extracellular signal-regulated kinases (ERK) [78] (See Fig. 4). Except for the three mechanisms mentioned above, DNA-dependent protein kinase catalytic subunit (DNA-PKcs) is also considered to be required for Sal-induced autophagy activation [18]. Table 1 has summarized the relevant information in recent advances.

**Table 1:** The induction or inhibition of autophagy triggered by Sal in vitro and in vivo models

Model	Induction		Inhibition		References
	+	Mechanisms	+	Mechanisms	
Colon cancer cell lines (RKO,SW480 and SW620) and breast cancer cell lines (MCF-7, T47D, and MDA –MB -453)	+	Sal activates the JNK pathway through generation of ROS			[40]
Osteoblastoma cells (U2OS and MG-63)	+	Sal activates the AMPK pathway through generation of ROS			[42]
Prostrate cancer cell line (PC3), breast cancer cell lines (SKBR3and MDAMB468) and murine embryonic fibroblast (MEF) cells	+	Sal leads to mitochondrial dysfunction with ATP -depletion, which activates the autophagy			[62]
Human NSCLC cell lines (A549, H460, Calu-1 and H157), human esophageal carcinoma cell line (TE3) and human pancreatic carcinoma cell line (PANC-1)	+	Sal induces ER stress -mediated autophagy via the ATF4 -DDIT3 /CHOP -TRIB3 -AKT1 -MTOR axis			[43]
Breast cancer cell line (MCF-7, HMLER and HMLER CD24 ^low/−^)			+	Sal inhibits the activity of lysosomal proteolytic.	[45]
Hepatocellular Carcinoma cell lines (HepG2 and Huh7) and primary human hepatocytes			+	Sal inhibits late stages of autophagy.	[47]
Human prostate cancer cell line (PC3), human breast cancer cell line (SKBR3)and murine embryonic fibroblast cells	+	/	+	/	[54]
Human prostrate cancer cell line (PC3),human primary dermal fbroblasts cells, tongue cancer cells (LK0412), larynx cancer cells (LK0923) and Normal Oral Keratinocytes (NOK)	+	Sal activates the AMPK pathway through generation of ROS.			[84]
Liver cancer cells (HepG2) and liver cancer stem cells (LCSCs)	+	/			[44]
Human prostrate cancer cell line (LNCaP, RWPE-1 and PC-3)	+	Sal inactivates mTORC1 and activates the AMPK pathway.			[41]
OS cells (U2OS and MG-63 lines)	+	DNA-PKcs is required for Sal-induced autophagy activation.			[18]
Glioblastoma cell lines (GSC23, GSC11, GSC7–2, GSC2–27, GSC5–22, GSC2, GSC231, GSC7–11, GSC10–6, GSC11–28 GSC229, SF188, KNS42, RES259, RES186, U87 MG, U373 MG, U251 MG and T98G)	+	Sal induces ER stress-mediated autophagy through generation of ROS.	+	Autophagic flux in Sal-treated cells is blocked by lipid oxidation due to oxidative stress, causing the increase of LMP.	[46]
Human osteosarcoma cell line (U2OS)	+	Sal triggers ROS-mediated autophagy.			[20]
Breast cancer cell lines (MCF-7and MDA-MB-231)	+	Sal triggers ROS-mediated autophagy.			[73]
Human glioma cell line (U87MG)	+	Sal induces ER stress-mediated autophagy through generation of ROS.			[75]

“/” indicates “not available”

### PI3K/AKT/mTOR signaling pathway

Two kind of mammalian target of rapamycin (mTOR) complexes, the mTOR complex 1 (mTORC1) and complex 2 (mTORC2), have been verified. The rapamycin-sensitive mTORC1 is composed of mTOR, regulatory associated protein of mTOR (Raptor), G protein β-subunit-like protein (GβL) and DEP domain-containing mTOR-interacting protein (DEPTOR). The mTOR-interacting protein can stabilize the mTOR dimer and promote mTOR’s substrate specificity [79–82]. In comparison, mTORC2, which is not as sensitive to rapamycin as the former, is composed of mTOR, GβL, rapamycin-insensitive companion of mTOR (Rictor), protein observed with RICTOR (Protor) and stress-activated protein kinase-interacting protein 1 (SIN1) [80, 81] mTORC1 obtains energy via small GTPases, for instance, Ras-related GTP binding protein (Rag) and Ras homolog enriched in brain (Rheb). Elevated level of amino acid and growth factors can activate mTORC1 through Rag and Rheb, separately. Activation of mTORC1 mainly lead to the phosphorylation of two downstream targets, p70 ribosomal S6 kinase/S6 ribosomal protein (p70S6K/S6RP) and eukaryotic translation-initiation factor 4E–binding protein 1 (4E–BP1), enhancing protein synthesis from increased ribosome biogenesis and messenger RNA translation under nutrient-rich conditions, promoting lipid biogenesis, and inhibiting autophagy [81].. The TSC2/TSC1 tuberous sclerosis complex, acting as downstream of AKT, negatively regulates mTORC1 by inhibiting the GTPase activity of Rheb, which is a positive regulator of mTORC1. TSC2/TSC1 inhibition due to AKT-mediated TSC2 phosphorylation at serine-939 and threonine-1462 leads to mTORC1 activation, whereas mTORC1 inhibition and consequent cell growth arrest in response to nutrient deprivation. Under nutrient-rich conditions, mTORC1 limits autophagy to counter lysosomal engulfment and degradation of cellular organelles and other cytosolic contents by mediating inhibitory phosphorylation of the unc-51-like kinase 1 (ULK1) complex, an obligatory component of autophagosome [83] .

A study has verified that Sal blocks inhibitory phosphorylation of TSC2 by AKT. Treatment of 1 μM Sal in the cells pre-treated with an Akt inhibitor, Triciribine, triggered increased accumulation of the autophagic marker LC3-II. However, under the same conditions in the presence of 10 μM Sal, it cannot observe any change in LC3-II levels compared to Sal treatment alone [84]. Sal’s blocked effect on AKT is further proved that reduced TSC2 phosphorylation at Ser-939 and Thr-1462 (two AKT-targeted sites) within 6 h Sal treatment contributes to mTORC1 inactivation [41]. Furthermore, Sal decreases phosphorylated of AKT and phosphorylated mTOR in prostate cancer cells. In addition, pretreatment with NAC, a general ROS scavenger, inhibits Sal-induced autophagy by suppressing ROS production [74].

### AMPK signaling pathway

AMPK activation dictates energy metabolism, gene transcription, cell mitosis and autophagy through regulating its many downstream kinases [85, 86]. In contrast to mTORC1, AMPK is a positive regulator of autophagy under the condition of nutrient/energy depletion. On the one hand, phosphorylation of TSC2 and raptor by AMPK is known to elevate TSC2/TSC1 activity and reduce mTOR’s kinase function, respectively [87, 88]. On the other hand, AMPK stimulates phosphorylation of the ULK1 complex, which is essential for the initiation of autophagy [89].

Sal induces a profound AMPK activation. AMPK inhibition by compound C or by AMPKα RNAi prevents Sal-induced autophagy activation. AMPK activation relies on ROS production in osteoblastoma cells, antioxidant NAC significantly inhibits Sal-induced AMPK activation and autophagic induction [42]. Further, another study has reported that AMPK activation and raptor phosphorylation are detected within 1 h after Sal treatment, whereas increased TSC2 phosphorylation at Ser-1387 and reduced TSC2 phosphorylation at Ser-939/Thr-1462 are detected after 2 h -- changes being more prominent at 20 h [41].

### MAPK signaling pathway

MAPK signaling pathway is generally considered to be a vital pathway to initiate the autophagic flux [90, 91].One way to activate the MAPK pathways is through the inactivation of MAPK-phosphatases, of which ROS can oxidize the catalytic site cysteine [92]. Another pathway for the activation of JNK proceeds via apoptotic signal-regulating kinase 1 (ASK1) [93]. This subsequently leads to the phosphorylation of JNK, and its target the transcription factor JUN. JNK activation may promote autophagy through the induction of ATG7 [94], or phosphorylation of BCL2, leading to dissociation of BCL2 from Beclin-1 [95].

Sal can lead to the formation of ROS eliciting JNK activation and induction of the transcription factor JUN. However, JNK inhibition can only partially block autophagy. Other signaling pathways must also play a role after administration of Sal [40]. Moreover, pretreatment with PD98059 and SB203580, an ERK and p38 inhibitors, suppresses the Sal-induced autophagy by reversing the upregulation of ERK and p38. Notably, Sal decreases the phosphorylation of AKT and the phosphorylation of mTOR in prostate cancer cells. In addition, pretreatment with NAC, an antioxidant, inhibits Sal-induced autophagy by suppressing ROS production. Furthermore, Sal activates ERK and p38 MAPK in a time-dependent manner in both PC-3 and LNCaP cells. Autophagy and LC3 expression are significantly restored by a ROS scavenger, NAC, in both PC-3 and LNCaP cells [74].

### ER stress

The endoplasmic reticulum (ER) is an organelle where synthesis, folding and maturation of proteins happen. Growing evidence indicate that the ER may act as a source of the membrane of autophagosomes [96, 97]. ER stress activates the unfolded protein response (UPR), which initiates the inhibition of protein translation through phosphorylation of eukaryotic translation initiation factor 2A (EIF2A) [98]. The phosphorylation of EIF2A stimulates the selective translation of transcription factors nuclear protein 1(NUPR1), activating transcription factor 4 (ATF4), and DNA damage inducible transcript 3(DDIT3) as well as the pseudokinase tribbles pseudokinase 3(TRIB3). The pseudokinase TRIB3 inhibits the AKT1-MTORC1 axis to stimulate autophagy [99, 100].

It has been demonstrated that Sal suppresses AKT1 activity through ATF4- DDIT3/CHOP- TRIB3- AKT1 axis in human non-small cell lung cancer (NSCLC) cells after activation of ER stress response, resulting in mTOR inhibition and autophagy consequently. Moreover, Sal upregulates ER stress-related proteins such as phospho-EIF2A, ATF4, DDIT3 in a time- and dose- dependent manner in human NSCLC cells. Furthermore, using RNA interference against ATF4 or DDIT3 and inhibitors of ER stress in combination with Sal, it has been confirmed that the EIF2A-ATF4-DDIT3 axis is the crucial mediator of Sal-induced autophagy. Besides, data have demonstrated that Sal reduces the activation of AKT1 as well as its downstream substrate mTOR, leading to autophagy. Similar study also has demonstrated that Sal induces ROS generation and the ROS scavenger NAC is found to inhibit the Sal-induced apoptosis, ER stress and autophagy. The inhibition of ER stress with 4-phenylbutyric acid depresses Sal-induced apoptosis and autophagy [75].

### Other mechanisms

In addition to the mainstream of autophagy activated pathways are mentioned above. DNA-PKcs is also required for Sal-induced autophagic activation [18]. DNA-PKcs is the 460-kDa serine/threonine protein kinase that belongs to PI3K-like protein kinase (PIKK) kinase family [101]. DNA-PKcs will be activated when facing DNA damages, and its normal function is to provoke non-homologous end joining (NHEJ) pathway to repair DNA double strand breaks [102]. DNA-PKcs inhibition, shRNA knockdown or miR-101 expression inhibits Sal-induced Beclin-1 expression and autophagic induction [18].

## Sal-induced cancer cell death via other cellular responses

It has been reported that Sal also induces apoptosis, which differs among the diverse cell types, in cancer cells [14, 16, 72, 103, 104] or different originated CSCs [7, 21, 105]. Apoptosis is induced in colorectal CSCs in a caspase-dependent manner, which proved by an increase of cleaved caspase-3, -8 and -9. JC-1 staining further revealed that Sal induces colorectal cancer cell apoptosis via the mitochondrial pathway [21]. Besides, Sal has been reported to induce human lung cancer cell apoptosis through the caspase 3/7-associated cell death pathway [106]. Moreover, Sal triggers apoptosis of PC-3 cells by increasing the intracellular ROS level, which is associated with reduced MMP, translocation of Bax protein to mitochondria, release of cytochrome c to the cytoplasm, activation of the caspase-3, cleavage of PARP-1 as well as the inhibition of the survival protein Bcl-2 [72]. It also has been noted that non-malignant RWPE-1 prostate cells are relatively less sensitive to Sal-induced lethality [72]. Besides the classical apoptotic pathway, Sal has been demonstrated to activate a unique apoptotic pathway that is not accompanied by cell cycle arrest and is independent of tumor suppressor protein p53, caspase activation, the CD95/CD95L system and the proteasome, which has been thought to overcome the high resistance to other anti-cancer drugs of cancer cells by increasing the expression of the Bcl-2 protein, P-gp as well as the 26S proteasome [16].

Uncontrolled cell cycle is an extremely important part of tumorigenesis. Several studies have also found that Sal can play a role in inhibiting cancer cell proliferation by inducing cell cycle arrest. It has been documented that Sal induces apoptosis in NCI/ADR-RES, DXR, and OVCAR-8 ovarian cancer cells via downregulation of S-phase kinase-associated protein-2 (Skp-2) and signal transducer and activator of transcription 3 (Stat3) inactivation. Skp-2 is overexpressed in majority of the CSCs. p27kip1 protein inhibits tumorigenesis through inhibiting cell cycle changes. Sal induces degradation of Skp2 and thus accumulated p27Kip1, ultimately cell cycle being arrested at G1 and apoptosis being induced [25]. Another study has showed that high concentrations of Sal induces a G2 arrest via downregulation cyclin B1, downregulation of survivin and triggering apoptosis. What is an interesting thing is that the administration with low concentrations of Sal induces a transient G1 arrest through downregulation cyclin D1 at an earlier time point and G2 arrest via downregulation cyclin B1 at a later point [107].

Autophagic cell death (ACD), named type II programmed cell death, distinguishes from apoptosis and necrosis, which was first established based on observations of increased autophagic markers in dying cells [108]. There is a big difference between ACD and apoptosis process. The morphological characteristics of apoptosis-related cells are the clearance of cytoskeleton and other proteins by caspases. Cytoskeletal clearance occurs in the early stages of apoptosis, but the organelles are cleared later; On the contrary, ACD is associated with a large accumulation of autophagic vacuoles in the cell, so that the degradation of organelles occurs at an early stage, and the cytoskeleton is cleared at a later stage [109]. In most cases, autophagy can serve as a cell survival promoter, however, autophagy may also act as cell death inducer. The pro-apoptotic effect of autophagy may result from two independent functions of autophagy: the pro-apoptotic function and the induction of ACD [108]. The autophagy regulator Atg5 activates caspase by interacting with the adaptor protein Fas-associated protein with death domain (FADD), a component of the extrinsic apoptotic pathway. Alternatively, cleavage of Atg5 by calpain causes the truncated Atg5 protein to translocate to the mitochondria to induce mitochondrial outer membrane permeabilization (MOMP) leading to cytochrome c release and caspase activation [109]. As a whole, there is a complicated relationship between autophagy and apoptosis. As mentioned above, Sal has been reported to induce cell death mainly via mitochondria-mediated apoptotic pathway, which can be considered as the executor of cancer cell death resulting from the severe damage caused by Sal. Whether the conjecture that the abnormal mitochondria in cancer cells or CSCs, which are not resistant to the effect of Sal, can be impaired by Sal and thus initiates the cell death process are true or not being worthy of further study. Moreover, several studies have showed that Sal both induces intensive autophagy and inhibits the late stage of autophagic flux which may play a crucial role in the ACD or pro-apoptotic process being of equal significance.

## Conclusion and perspective

Sal, being a K^+^ ionophore, can effectively target CSCs, kill chemo-resistant cancer cells and sensitize radio-resistant cancer cells simultaneously. Of note, its side effects are much less than the conventional chemotherapeutical drugs. Although Sal exhibits predominant anticancer effects in vivo and in vitro experiments and a small number of clinical trials, the exact mechanisms are still unknown.

We summarize the autophagy-related researches, providing a new sight into Sal’s preference for CSCs and cancer cells. Cancer cells are deemed to possess abnormal mitochondria, which may be not resistant to ion fluctuations caused by Sal, then leading to the dysfunction of organelles and the production of ROS, which initiates autophagy. Similarly, DCA, which is likewise a K^+^ ion channel modulator, preferentially targets cancer cells. Meanwhile, Sal can inhibit the late stage of the autophagic flux, which leads the accumulation of dysfunctional organelles and produces excessive ROS. The combined effects of these two processes lead to the death of tumor cells. The combination of a K^+^ ionophore and autophagic inhibitor may be able to confirm the rationality of this conjecture.

In addition, some studies have showed that the combined use of Sal and CQ, which is an autophagy inhibitor, not only improves antitumor effects of Sal but also reduces the dose of Sal [44]. Whereas, Sal is able to inhibit the late stage of autophagic flux, so whether the combined use of an early stage inhibitor or a late stage inhibitor can amplify the anticancer effects of Sal requires further studies. What’s more, another study has found that co-treatment with an inhibitor of the mitochondrial NCX can significantly reduce structural damage in the peripheral nervous system without impairing Sal’s anticancer efficacy in vivo [65]. Therefore, a large number of further studies should be carried out to provide a basis for future rational combinative therapy.

CSCs are generally considered to be the chief culprit for the initiation, recurrence and metastasis of cancer. Sal preferentially targeting CSCs may bring us closer to find a cure against this devastating disease. Generally speaking, specific and more potent toxicity of Sal towards CSCs and cancer cells without much adverse effect towards normal cells promise Sal may be an effective anticancer agent in the near future. Understanding the biological mechanism of Sal inducing cell death can aid in designing more effective and less toxic therapeutic strategies. As a new anticancer compound, whether Sal could eventually enter into clinical application needs the joint efforts of all researchers. Anyway the in-depth study of Sal’s direct targets or related signaling pathways will open a new chapter in the near future against cancers.

## Abbreviations

ACD: Autophagic cell death
AMPK: AMP-activated protein kinase
ASK1: Apoptosis signal-regulating kinase 1
ATF4: Activating transcription factor 4
AVO: Acidic vesicular organelles
CQ: Chloroquine
CSCs: Cancer stem cells
DCA: Dichloroacetate
DDIT3: DNA damage inducible transcript 3
DEPTOR: DEP domain-containing mTOR-interacting protein
DNA-PKcs: DNA-dependent protein kinase catalytic subunit
DRGNs: Dorsal root ganglia neurons
Drp-1: Dynamin-related protein
EIF2A: Eukaryotic translation initiation factor 2A
ER: Endoplasmic reticulum
ERK: Extracellular signal-regulated kinases
FADD: Fas-associated protein with death domain
GβL: G protein β-subunit-like protein
HCC: Hepatocellular Carcinoma
Hh: Hedgehog
JNK: C-jun N-terminal kinase
MAPK: Mitogen-activated protein kinase
Mfn1: Mitofusin 1.
Mfn2: Mitofusin 2.
MMP: Mitochondrial membrane potential.
MOMP: mitochondrial outer membrane permeabilization.
mTOR: Mammalian target of rapamycin.
NAC: N-acetyl-L-cysteine.
NCXs: Na^+^/ Ca^2+^ exchangers.
NHEJ: Non-homologous end joining.
NSCLC: Non-small cell lung cancer.
NUPR1: Nuclear protein 1.
OPA1: Optic atrophy protein 1.
pHe: Extracellular pH.
PIKK: PI3K-like protein kinase.
Protor: protein observed with RICTOR.
p70S6K: p70 ribosomal S6 kinase.
Raptor: regulatory associated protein of mTOR.
Rag: Ras-related GTP binding protein.
Rheb: Ras homolog enriched in brain.
Rictor: rapamycin-insensitive companion of mTOR.
ROS: Reactive oxygen species.
Sal: Salinomycin.
SIN1: stress-activated protein kinase-interacting protein 1.
Skp-2: S-phase kinase-associated protein-2.
Stat3: signal transducer and activator of transcription 3.
S6RP: S6 ribosomal protein.
TRIB3: Tribbles pseudokinase 3.
ULK1: Unc-51-like kinase 1.
UPR: Unfolded protein response.
4E–BP1: 4E–binding protein 1.
